# Supplementary material for: Co-Zn-MOFs Derived N-Doped Carbon Nanotubes with Crystalline Co Nanoparticles Embedded as Effective Oxygen Electrocatalysts
Source: Nanomaterials (Basel). 2021 Jan 20;11(2):261. doi: 10.3390/nano11020261 (PMC7909561; doi:10.3390/nano11020261)
Supplement: Supplementary file 1 [file nanomaterials-11-00261-s001.pdf]

Co-Zn-MOFs derived N-doped carbon nanotubes with crystalline Co nanoparticles embedded as effective oxygen electrocatalysts

Wendi Zhang,[a] Xiaoming Liu, [a] Man Gao, [a] Hong Shang, [a] and Xuan-He Liu*[a]

[a] School of Science, China University of Geosciences (Beijing), Beijing 100083, People's Republic of China.

E-mail address: liuxh@cugb.edu.cn (X. H. Liu)

Chemical reagents

1,4-benzenedicarboxylic acid (H_2BDC, >99.5%), N,N-Dimethylformamide (DMF, > 99.8%), ethanol, $CoCl_2 \cdot 6H_2O$ (>98.0%), $ZnCl_2$ (>99.5%), triethylamine (TEA, >98.5%), Dicyandiamide (DCD, >99.5%), Carbon black, Commercial Pt/C, blank CNTs (>99.9%) , Nafion (5% w/w water and 1-propanol ethyl carbinol). All chemical reagents were bought from Aladdin Reagent.

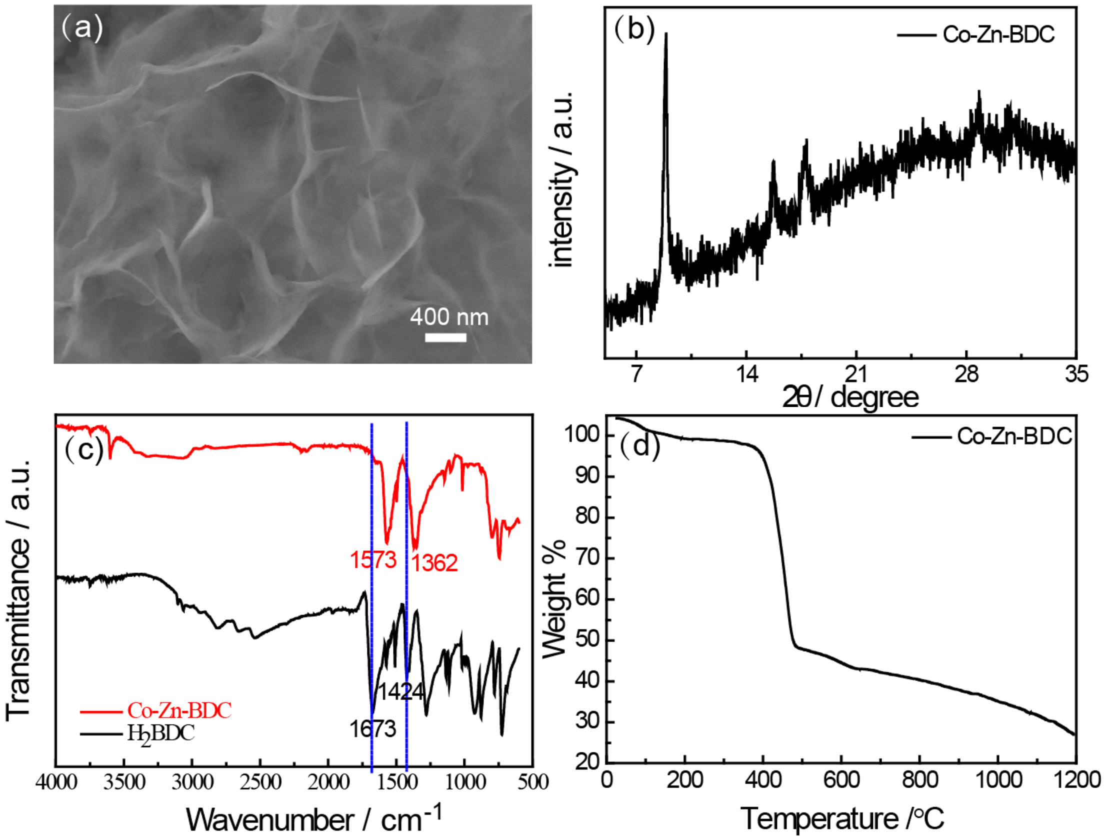

Figure S1 **(a)** SEM image of Co-Zn-BDC; **(b)** XRD pattern of Co-Zn-BDC; **(c)** FTIR spectra of Co-Zn-BDC (red) and H_2BDC (black); **(d)** Thermogravimetric analysis of Co-Zn-BDC.

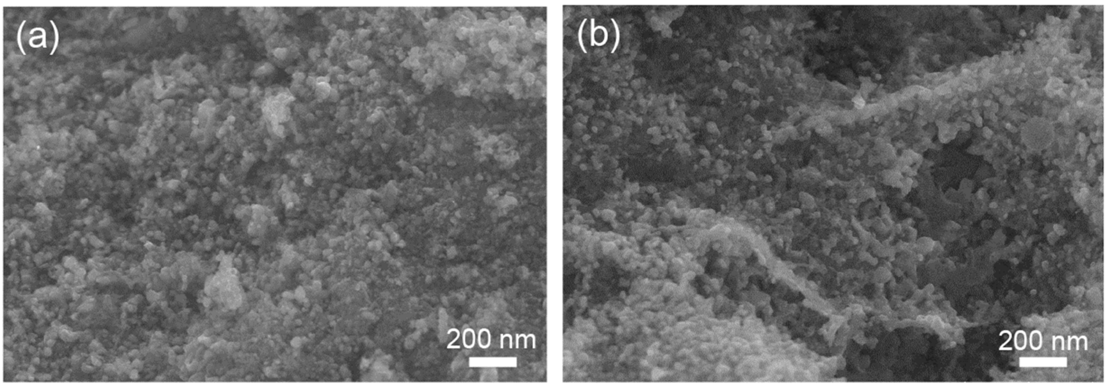

Figure S2 **(a)** SEM image of Co@CNTs-800 (Co-Zn-BDC：DCD =1:2); **(b)** SEM image of Co@CNTs-800 (Co-Zn-BDC：DCD =2:1).

Table S1. The content of N, C and H for Co@CNTs-600, Co@CNTs-700, Co@CNTs-800 and Co@CNTs-900 tested by Organic Element Analyzer.

Name	N (%)	C (%)	H (%)
Co@CNTs-600	2.75	15.67	0.69
Co@CNTs-700	3.01	37.78	0.21
Co@CNTs-800	1.87	45.53	0.22
Co@CNTs-900	1.25	53.34	0.16

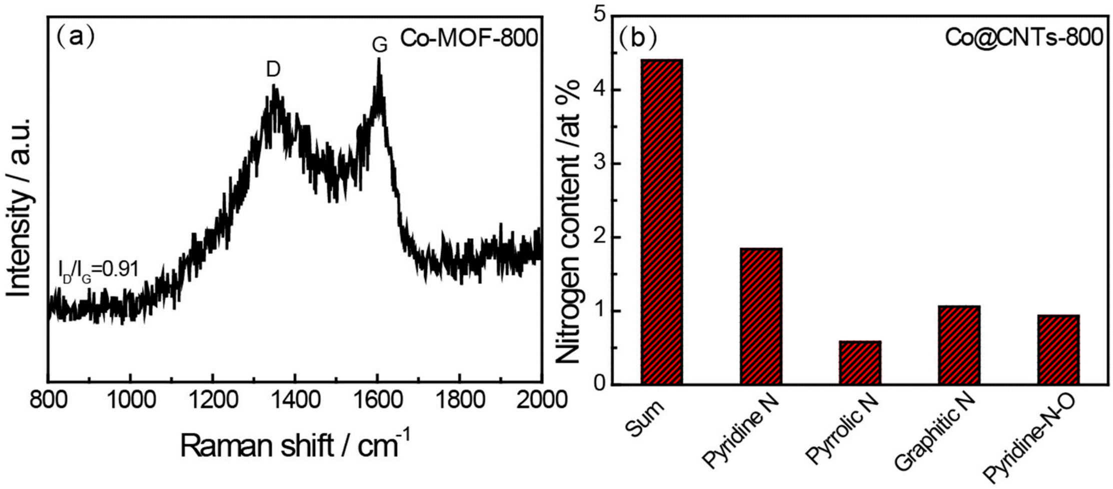

Figure S3 **(a)** Raman spectra of Co-MOF-800; **(b)** contents of various N species in Co@CNTs-800.

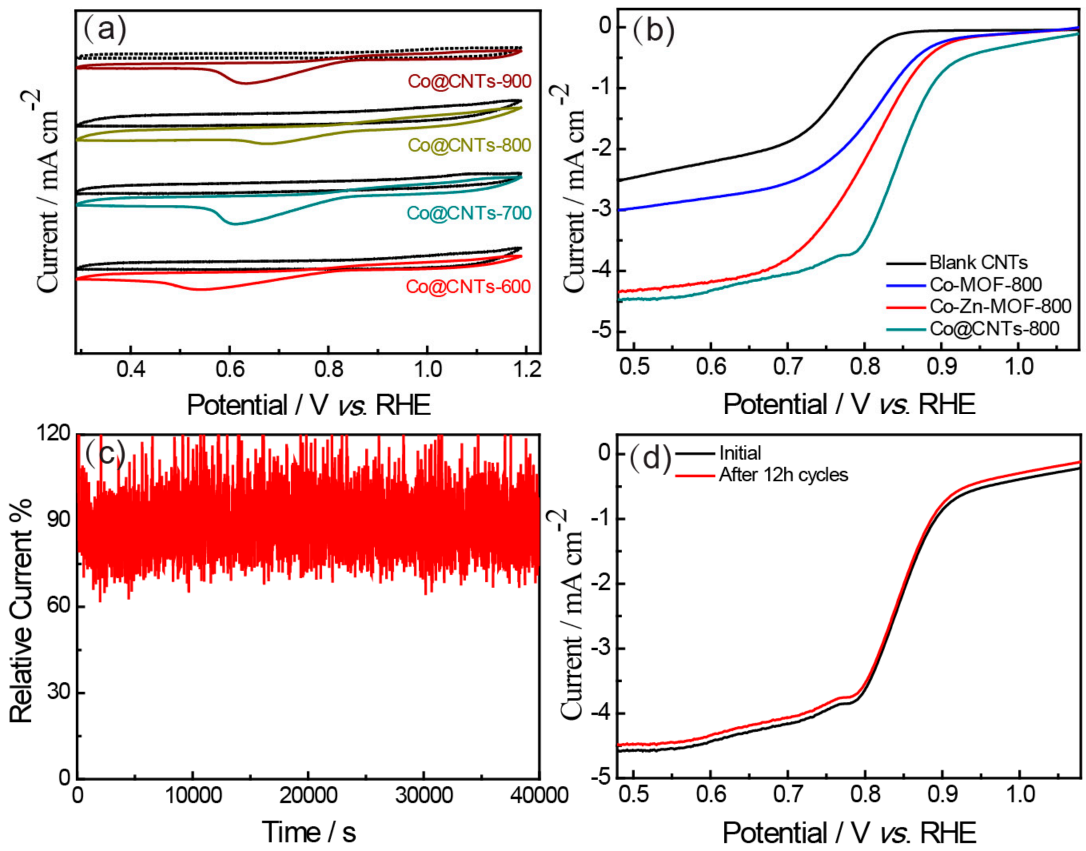

Figure S4 **(a)** CV curves of Co@CNTs-T catalyst at 600,700, 800 and 900 ºC in N_2 and O_2-saturated 0.1 M KOH solution, respectively; **(b)** LSV curves of blank CNTs, Co-MOF-800, Co-Zn-MOF-800 and Co@CNTs-800; **(c)** Chronoamperometric responses for Co@CNTs-800 in O_2-saturated electrolyte solution at -0.30 V (vs. Ag/AgCl); **(d)** LSV curves of Co@CNTs-800 of initial and after 12 h test.

Table S2 The comparison of ORR activity of Co@CNTs-800 with the previously reported non-precious ORR electrocatalysts.

catalyst	Electrolyte	Half-wave Potential (vs. RHE)	Electron transfer number	Limit current [mA cm^{-2}]	Ref.
Co@CNTs-800	0.1M KOH	0.85 V	3.94	5.0	This work
L-CCNTs-Co-800	0.1M KOH	0.84 V	3.87	5.1	1
(Co,Zn)/N–C	0.1M KOH	0.86 V	3.88	6.1	2
Fe/OES	0.1M KOH	0.85 V	3.8-4.0	6.1	3
3D mesoporous Fe-C-N	0.1M KOH	0.84 V	>3.92	6.5	4

CoNC‑CNF-1000	0.1M KOH	0.80 V	3.96	5.9	5
FeCo-NC	0.1M KOH	0.84 V	3.8	5.3	6
B, N, P and Fe-doped Graphene Foams	0.1M KOH	0.84 V	3.8	5.8	7
3D Co–N-C	0.1M KOH	0.81 V	3.75	5.3	8
Co-N carbon tubes	0.1M KOH	0.87 V	3.85-3.88	4.2	9
Fe/N/P Co-Doped 3D Porous Carbon	0.1M KOH	0.84 V	3.2	4.5	10
Fe/Fe_3C N-Doped Hollow Carbon Spheres	0.1M KOH	0.84 V	3.88-3.98	6.0	11
Co-N-C/CNTs	0.1M KOH	0.81 V	3.96	5.2	12

[1] Liang, Z.; Fan, X.; Lei, H.; Qi, J.; Li, Y.; Gao, J.; Huo, M.; Yuan, H.; Zhang, W.; Lin, H.; Zheng, H.; Cao, R. Cobalt–Nitrogen‐Doped Helical Carbonaceous Nanotubes as a Class of Efficient Electrocatalysts for the Oxygen Reduction Reaction. *Angew. Chem. Int. Ed.* **2018**, *130*, 13371-13375.

[2] Lu, Z.; Wang, B.; Hu, Y.; Liu, W.; Zhao, Y.; Yang, R.; Li, Z.; Luo, J.; Chi, B.; Jiang, Z.; Li, M.; Mu, S.; Liao, S.; Zhang, J.; Sun, X. An isolated zinc–cobalt atomic pair for highly active and durable oxygen reduction. *Angew. Chem. Int. Ed.* **2019**, *131*, 2648-2652.

[3] Hou, C. C.; Zou, L.; Sun, L.; Zhang, K.; Liu, Z.; Li, Y.; Li, C.; Zou, R.; Yu, J.; Xu, Q. Single‐Atom Iron Catalysts on Overhang‐Eave Carbon Cages for High‐Performance Oxygen Reduction Reaction. *Angew. Chem. Int. Ed.* **2020**, *132*, 7454-7459.

[4] Liu, S.; Yang, Z.; Li, M.; Lv, W.; Liu, L.; Wang, Y.; Chen, X.; Zhao, X.; Zhu, P.; Wang, G. Facile synthesis of 3D hierarchical mesoporous Fe-CN catalysts as efficient electrocatalysts for oxygen reduction reaction. *Int. J Hydrogen Energ*. **2018**, *43*, 5163-5174.

[5] Zhang, W.; Yao, X.; Zhou, S.; Li, X.; Li, L.; Yu, Z.; Gu, L. ZIF-8/ZIF-67-Derived Co-Nx-Embedded 1D Porous Carbon Nanofibers with Graphitic Carbon-Encased Co Nanoparticles as an Efficient Bifunctional Electrocatalyst. *Small*. **2018**, *14*, 1800423.

[6] Zhong, B.; Zhang, L.; Yu, J.; Fan, K. Ultrafine iron-cobalt nanoparticles embedded in nitrogen-doped porous carbon matrix for oxygen reduction reaction and zinc-air batteries. *J. Colloid. Interf. Sci*. **2019**, *546*, 113-121.

[7] Dong, F.; Cai, Y.; Liu, C.; Liu, J.; Qiao, J. Heteroatom (B, N and P) doped porous graphene foams for efficient oxygen reduction reaction electrocatalysis. *Int. J Hydrogen Energ.* **2018**, *43*, 12661-12670.

[8] Chen, S.; Cheng, J.; Ma, L.; Zhou, S.; Xu, X.; Zhi, C.; Zhang, W.; Zhi, L.; Zapien, J. A. Light-weight 3D Co–N-doped hollow carbon spheres as efficient electrocatalysts for rechargeable zinc–air batteries. *Nanoscale*, **2018**, *10*, 10412-10419.

[9] Zhou, Q.; Zhang, Z.; Cai, J.; Liu, B.; Zhang, Y.; Gong, X.; Sui, X.; Yu, A.; Zhao, L.; Wang, Z.; Chen, Z. Template-guided synthesis of Co nanoparticles embedded in hollow nitrogen doped carbon tubes as a highly efficient catalyst for rechargeable Zn-air batteries. *Nano Energy*, **2020**, *71*, 104592.

[10] Liu, J.; Zhu, Y.; Du, F.; Jiang, L. Iron/Nitrogen/Phosphorus Co-Doped Three-Dimensional Porous Carbon as a Highly Efficient Electrocatalyst for Oxygen Reduction Reaction. *J. Electrochem. soc.* **2019**, *166*, F935.

[11] Liu, Y.; Wang, X.; Zhao, B.; Shao, X.; Huang, M. Fe/Fe_3C Nanoparticles Encapsulated in N-

Doped Hollow Carbon Spheres as Efficient Electrocatalysts for the Oxygen Reduction Reaction over a Wide pH Range. *Chem–Eur. J.* **2019**, *25*, 9650-9657.
[12] Guo, H.; Feng, Q.; Zhu, J.; Xu, J.; Li, Q.; Liu, S.; Xu, K.; Zhang, C.; Liu, T. Cobalt nanoparticle-embedded nitrogen-doped carbon/carbon nanotube frameworks derived from a metal–organic framework for tri-functional ORR, OER and HER electrocatalysis. *J. Mater. Chem. A.* **2019**, *7*, 3664-3672.
